# Metabolism in tumor microenvironment: Implications for cancer immunotherapy

**DOI:** 10.1002/mco2.6

**Published:** 2020-06-03

**Authors:** Rongchen Shi, Yi‐Quan Tang, Hongming Miao

**Affiliations:** ^1^ Department of Biochemistry and Molecular Biology Third Military Medical University (Army Medical University) Chongqing People's Republic of China; ^2^ MRC Laboratory of Molecular Biology Cambridge Biomedical Campus Cambridge UK

**Keywords:** cancer therapy, immunity, metabolism, tumor microenvironment

## Abstract

Tumor microenvironment is a special environment for tumor survival, which is characterized by hypoxia, acidity, nutrient deficiency, and immunosuppression. The environment consists of the vasculature, immune cells, extracellular matrix, and proteins or metabolic molecules. A large number of recent studies have shown that not only tumor cells but also the immune cells in the tumor microenvironment have undergone metabolic reprogramming, which is closely related to tumor drug resistance and malignant progression. Tumor immunotherapy based on T cells gives patients new hope, but faces the dilemma of low response rate. New strategies sensitizing cancer immunotherapy are urgently needed. Metabolic reprogramming can directly affect the biological activity of tumor cells and also regulate the differentiation and activation of immune cells. The authors aim to review the characteristics of tumor microenvironment, the metabolic changes of tumor‐associated immune cells, and the regulatory role of metabolic reprogramming in cancer immunotherapy.

## Graphical abstract

Metabolic reprogramming is the basis of tumor microenvironment, which consists of cellular and extracellular components. The cellular components are mainly composed of hematopoietic immune cells (For example, TAMs, tumor‐associated macrophages; TILs, tumor‐infiltrated lymphocytes; TADCs, tumor‐associated dendritic cells; TANKs, tumor‐associated natural killer cells) and resident stromal cells. The extracellular components are mainly composed of extracellular matrix and cell‐secreted factors. The interaction between cancer cells and interstitial cells in the tumor microenvironment regulates tumorigenesis and progression.

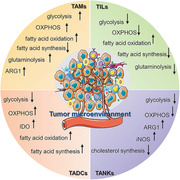

## 1 INTRODUCTION

The transformation from normal cells to neoplastic cells is widely believed to be due to DNA mutations, which causes loss of susceptibility to the tumor immune microenvironment.^1^ Under normal circumstances, such cells are quickly cleared by the immune system. However, the failure of immunosurveillance leads to the progression from neoplasia to cancer.^2^, ^3^, ^4^ During this process, tumor cells evade the recognition and elimination of the immune system by regulating their own antigen processing and presentation machinery. Finally, cancerous cells continue to mutate in order to continuously escape immune surveillance, and eventually form tumors.^5^

**FIGURE 1:**
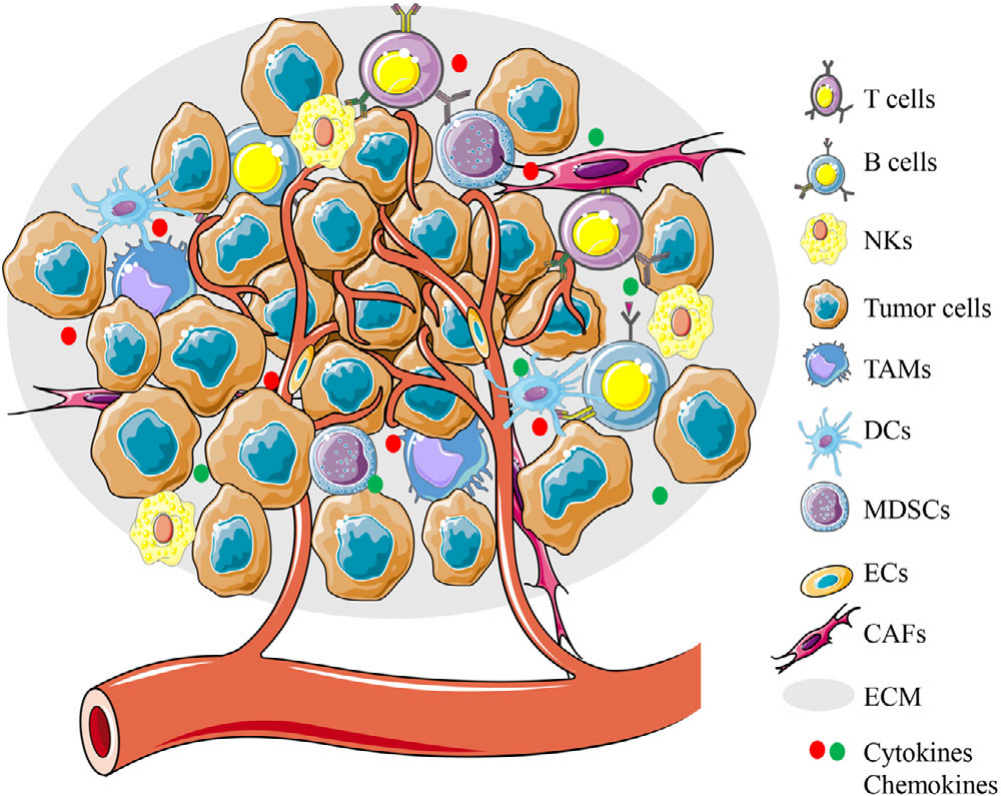
Components of the TME. The TME consists of cellular and extracellular components. The cellular components are mainly composed of hematopoietic immune cells (TAMs, T cells, B cells, NK cells, DCs, and MDSCs) and resident stromal cells (CAFs, ECs, etc). ECM and cell‐secreted proteins such as cytokines and growth factors constitute the extracellular components. The interaction between cancer cells and interstitial cells in the TME regulates tumorigenesis and progression Abbreviations: CAFs, cancer‐associated fibroblasts; DCs, dendritic cells; ECs, endothelial cells; ECM, extracellular matrix; MDSCs, myeloid‐derived suppressor cells; NKs, natural killer cells; TAMs, tumor‐associated macrophages.

Actually, cancer cells do not simply evade the immune surveillance by themselves. Cancer cells can also create an immunosuppressive microenvironment to regulate surrounding cells, which not only facilitates tumor growth, but also further promotes tumor immune escape.^6^ This microenvironment also called “Tumor Microenvironment” (TME)^7^ (Figure 1). The TME refers to the networks of cells and structures that surround tumor cells. Apart from the tumor cells, the TME includes surrounding vasculature, the extracellular matrix, other nonmalignant cells (immune cells, cancer‐associated fibroblasts, etc), and signaling molecules (cytokines, growth factors, hormones, etc.).^7^ The TME is not only closely related to the occurrence, growth, and metastasis of tumors, but also has a great impact on the treatment of tumors.^6^, ^8^, ^9^ In this review, we focus on the characteristics and composition of the TME, and summarize the metabolic changes of immune cells in the TME and their effects on cancer immunotherapy.

## 2 CHARACTERISTICS OF THE TUMOR MICROENVIRONMENT

**FIGURE 2:**
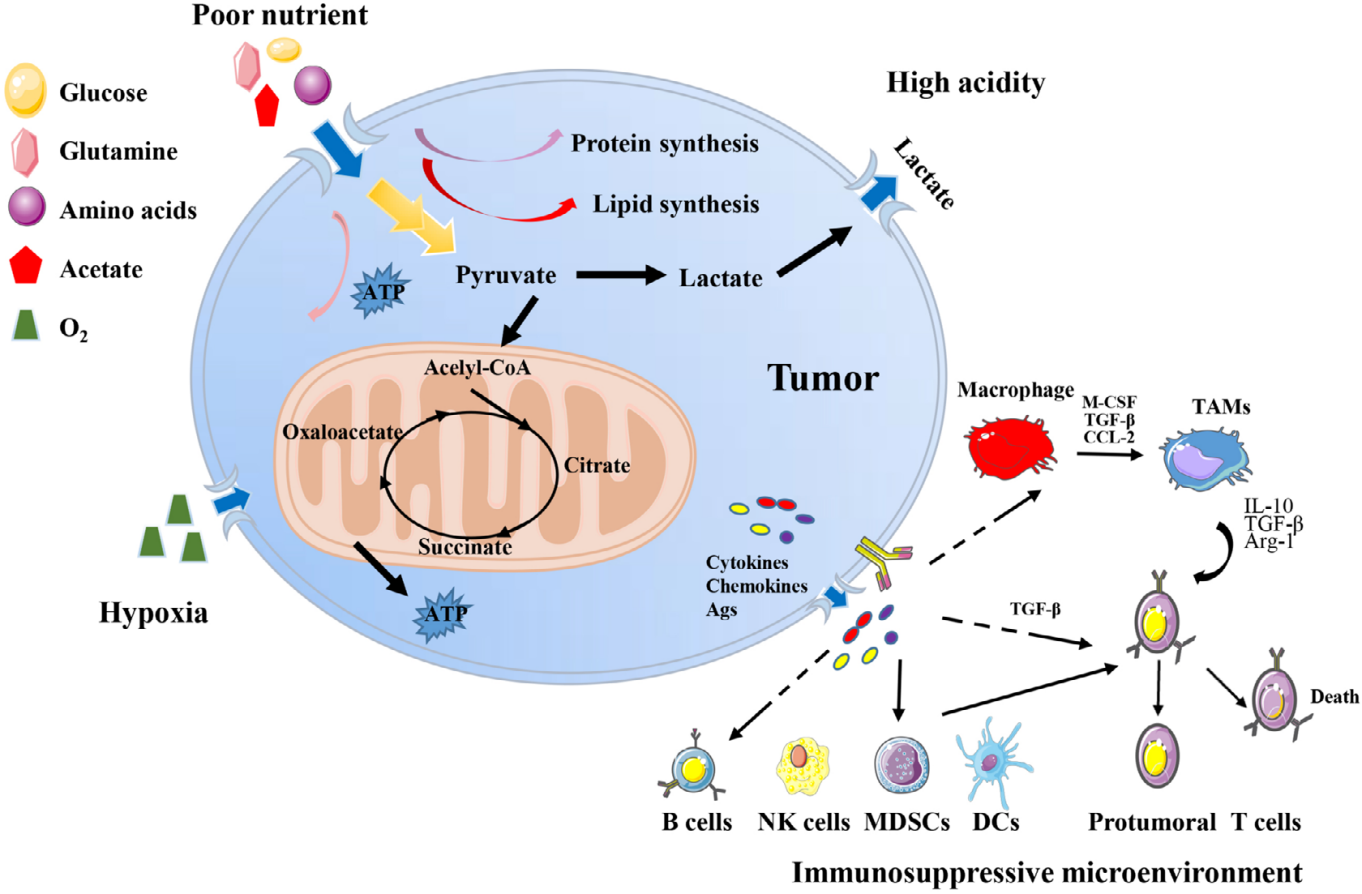
Characteristics of the TME. Tumor cells uptake a large sum of nutrients (glucose, amino acids, etc) from TME for substance and energy demands, resulting a nutrient‐poor environment. Cancer cells take aerobic glycolysis to produce and release lactic acid, making an acidity environment. Tumor cell‐derived cytokines, chemokines, metabolites, and antigens reprogram interstitial immune cells, forming an immunosuppressive environment, characterized by M2‐like TAMs, Tregs, MDSCs, and so forth. Rapid proliferation of tumor cells and the immaturity of tumor vasculature lead to hypoxia

The formation of the TME mainly depends on tumor metabolism.^10^ A common feature of tumor metabolism is in order to further consolidate their advantages, tumor cells competitively plunder the nutrients in the microenvironment, and finally promote the tumor malignant progression.^11^, ^12^ In this perspective, we summarize the characteristics of the TME into the following four aspects: (a) poor nutrient, (b) high acidity, (c) hypoxia, and (d) immunosuppressive microenvironment (Figure 2). Almost all TME have the above four characteristics, and investigation of the antecedents and consequences of the formation of the TME under these features may advance tumor research and improve clinical treatment.

### 2.1 Poor nutrient

In order to maintain their energy requirements related to survival and progression, cancer cells must continuously acquire nutrients from the TME, including two basic nutrients for tumor survival: glucose and glutamine.^13^ German physiologist, Otto Heinrich Warburg, firstly discovered that cancer cells perform glycolysis to provide energy even in the presence of sufficient oxygen, also known as the Warburg effect.^14^ Detection of the uptake of a radioactive fluorine‐labeled glucose analog, 18F‐fluorodeoxyglucose (18F‐FDG), by cells based on the Warburg effect has been successfully used in clinic.^15^ In the 1950s, American scientist Harry for the first time discovered the high demand of glutamine for the proliferation of cancer cells.^16^ Glutamine plays an important role in the biosynthesis of nucleotides, glucosamine‐6‐phosphate, and amino acids.^17^, ^18^ And 18F‐labeled glutamine tracer has also recently be shown to be promising in preclinical and early clinical studies, especially for brain tumors in which the use of 18F‐FDG is not feasible.^19^, ^20^ In addition, cancer cells have high demand for almost all nutriments, including lipids and amino acids in the TME,^13^ leading to a lack of sufficient nutrition for tumor interstitial cells. These changes may ultimately promote the malignant growth and proliferation of tumors.^21^ It has been demonstrated that insufficient glucose in the TME impairs T cell antitumor activity.^22^, ^23^, ^24^, ^25^, ^26^ Low‐glycemic tumor microenvironments have been found to decreased T cell viability, which are associated with the low expression of zeste methyltransferase enhancer homolog 2 (EZH2) and decreased glycolytic function.^27^ Researchers also found that the glycolytic rate of natural killer (NK) cells was low in the microenvironment of lung cancer, which further weakened its cytotoxicity and cytokine production.^28^

### 2.2 High acidity

Tumor cells take up a large amount of glucose in the TME for aerobic glycolysis to generate energy for their own use.^29^ Meanwhile, it also secretes a large amount of lactic acid, resulting in a hypoglycemic and acidic TME.^30^, ^31^ Low PH in the TME impairs the function of tumor mesenchymal cells, especially immune cells, leading to immunosuppression.^32^, ^33^ For example, lactic acids promote tumor‐associated macrophage M2 polarization and tumor malignant progression through the lactate‐MCT‐HIF1α axis as a critical signaling cascade.^34^ The low‐pH condition of the TME prevents the regeneration of NAD+, which acts as an important reductive equivalent, in T cells, and push the mitochondrial tricarboxylic acid cycle (TCA) forward to produce ATP, ultimately inhibiting the function of T cells and cytokines production.^31^ Recently, Gao et al proposed a novel intracellular/extracellular lactic acid depletion strategy, also called PMLR nanosystem. They constructed a hollow, MnO_2_‐catalyzed nanosystem loaded with the lactate oxidase and a glycolytic inhibitor, and coated with red blood cell membrane for lactate consumption and synergy antitumor metabolism and immunity therapy.^35^ PMLR nanosystem effectively inhibits tumor growth by blocking ATP production, and also significantly depletes lactic acid in TME, thus activating natural immunity and in situ cellular immunity.^35^ Importantly, this effective TME regulation strategy activates local tumor immunity to improve the therapeutic effect of checkpoint blocking therapy, and overcomes the systemic toxicity caused by immunoagonists.^35^

### 2.3 Hypoxia

Oxygen is necessary for cell metabolism to regulate biochemical reactions within cells.^36^ The rapid and uncontrolled proliferation of tumors leads to inadequate blood supply and hypoxia in TME, a characteristic of almost all solid tumors.^36^, ^37^, ^38^ Hypoxia generally refers to the area with the oxygen levels less than 2%.^39^ Although the rapid proliferation of tumors stimulates angiogenesis, the irregular distribution of new tumor blood vessels causes imbalanced tissue oxygenation capacity of capillaries, eventually leading to the generation of permanent or temporary hypoxic areas of the tumor.^40^ As tumors growing, they gradually adapt to hypoxia by utilizing hypoxia‐inducible factor 1α (HIF‐1α) to resist chemotherapy, radiotherapy, or immunotherapy.^41^ In the meanwhile, tumors develop a more aggressive and treatment‐resistant phenotype, which is closely related to the poor prognosis of patients.^41^, ^42^ In addition, hypoxia in the TME also affects the surrounding cells, especially the immune cells.^43^ Hypoxia not only promotes the death of immune effector cells and reduces the generation and release of cytokines (such as CD8^+^T cells^44^ and NK cells^45^, ^46^), but also supports the generation of immunosuppressive cells (including regulatory T [Treg] cells^47^ and M2‐like macrophages^34^, ^43^) and promotes the production and secretion of immunosuppressive cytokines. Studies have shown that reducing hypoxia by breathing high oxygen can promote the increase of T cell infiltration and the secretion of pro‐inflammatory cytokines, which can improve tumor regression and survival in mice.^48^ Recently, Wu et al found that hypoxia in the TME promotes the enrichment of triggering receptor expressed on myeloid cells‐1 (TREM‐1)^+^ macrophages in tumors, recruits C‐C Motif Chemokine Receptor 6 (CCR6)^+^ Treg cells through C‐C Motif Chemokine Ligand 20 (CCL20), and indirectly leads to the dysfunction and apoptosis of CD8^+^T cells, ultimately resulting in the formation of immunosuppressive microenvironment and resistance of PD1 blockade.^49^

### 2.4 Tumor immunosuppressive microenvironment

The formation of tumor immunosuppressive microenvironment depends on the coordination of multiple immune cells, including M2‐like tumor‐associated macrophages (TAMs),^50^ Treg cells^51^ myeloid‐derived suppressor cells (MDSCs),^52^ and so on. The chemokine CCL2 released during tumor growth promotes the infiltration of a large number of monocytes into the tumor by acting on the monocyte surface receptor CCR2.^53^ Interleukin‐4 (IL‐4) secreted by tumor cells and CD4^+^ T cells polarizes TAMs to an M2 phenotype and enhances tumor cell growth, invasion, and metastasis.^50^ TAMs also express a series of immune suppressor molecules and promote the formation of an immunosuppressive microenvironment. For example, TAMs express the major histocompatibility complexes HLA‐E and HLA‐G, which bind to CD94 and Ig‐like transcript 2 (ILT2) on the surface of NK cells and T cells, respectively, and inhibit their activation.^54^ Additionally, TAMs secrete cytokines such as IL‐10 and transforming growth factor‐β (TGF‐β) to inhibit the activity of CD4^+^ T cells and CD8^+^ T cells and mediate the proliferation of Treg cells, and finally maintain the tumor immunosuppressive microenvironment.^55^ Moreover, vascular endothelial growth factor (VEGF), granulocyte‐macrophage colony‐stimulating factor (GM‐CSF), TGF‐β, CCL2, interferon gamma (IFN‐γ), IL‐6, IL‐10, and IL‐1β in the TME also promote MDSC infiltration into tumors. MDSCs produce high levels of nitric oxide (NO), arginine (Arg)‐1, and IL‐10, which inhibit antigen‐specific and nonspecific T cell responses. The MDSCs also promote the recruitment of Treg cells by secreting CCL3, CCL4, and CCL5.^52^, ^56^, ^57^ Taken together, in the TME, along with the great suppression of immune killer cells, recruitment and activation of immunosuppressive cells is conducive to the immune escape of tumor cells and promotes tumor progression.

## 3 MAJOR IMMUNE CONSTITUENTS OF THE TUMOR MICROENVIRONMENT

### 3.1 Tumor‐associated macrophages

TAMs account for the largest proportion (up to 50%) of myeloid infiltrate in most human solid malignancies.^50^ The high heterogeneity of TAMs is not only found in different cancer patients, but also in different malignant lesions and specific tumor lesions of the same patient.^58^ In the TME, especially in malignant tumors with a high degree of progression, most TAMs tend to be M2 polarized, which is closely related to tumor growth, invasion, and metastasis; the construction of an immunosuppressive microenvironment; and the poor prognosis of patients.^59^, ^60^ However, little is known about how TME regulates metabolic changes in TAMs. Here, we review the metabolic changes of TAMs and their possible applications in the treatment of malignant tumors.

### 3.2 Glucose metabolism

To date, it is generally believed that proinflammatory M1 macrophages are mainly characterized by enhanced glycolysis and attenuated TCAs, and are generally considered to have strong antitumor activity.^61^ Anti‐inflammatory M2 macrophages, however, show complete TCA and enhanced fatty acid oxidation, which promote tumor progression.^50^ Interestingly, TAMs enhance aerobic glycolysis, and exhibit a mixed phenotype of M1 and M2, with increased expression of pro‐inflammatory cytokines and chemokines, Arg1, IL4Ra, and M2 macrophage‐associated cytokines, which may be mediated to some extent by tumor‐derived lactate via Akt/mTOR signaling.^62^ TAMs promote blood vessel formation, enhance tumor cell extravasation, and induce epithelial‐mesenchymal transition, ultimately causing tumor invasion and metastasis, which could be disrupted by the inhibition of glycolysis with 2‐deoxyglucose.^58^ However, mTOR inhibition as a therapeutic target in cancer weakens TAM glycolysis in the hypoxic area, causing abnormal vascular structure and further promoting metastasis, which limits the use of this therapy.^63^ In contrast, it's also reported that oxidative phosphorylation is elevated in thyroid carcinoma‐primed macrophages through measurement of oxygen consumption rates, suggesting that oxidative phosphorylation is still intact despite of a broken TCA.^64^ Given the high heterogeneity of glucose oxidation in TAMs, more specific studies on the TAM subsets are needed in future.

### 3.3 Lipid metabolism

Activated macrophages also exhibit changes in lipid metabolism. M2 macrophages usually show strong fatty acid oxidation, which may be driven by activation of signal sensors, such as transcriptional activator 6 and peroxisome proliferator‐activated receptor gamma coactivator‐1 beta (PGC‐1β) in response to IL‐4 treatment.^65^ IL‐4‐activated macrophages exhibit increased triglyceride uptake through CD36 and enhanced FAO to supply cellular energy.^66^, ^67^ In contrast, lipopolysaccharide‐stimulated macrophages show enhanced synthesis of fatty acid and triglyceride, with an induction of pro‐inflammatory cytokines.^68^ In addition, enhancement of FAS is required for the augment of phagocytosis in monocytes.^69^

In the TME, TAMs also change their lipid metabolism to adapt to environmental changes. TAMs enhance the biosynthesis, uptake, or storage of fatty acids, and exert the pro‐ or antitumor effects depending on special lipid metabolism‐associated cytokine prodiction.^70^, ^71^ For example, TAMs highly express epidermal fatty acid binding protein (E‐FABP), which promotes the formation of lipid droplets and IFN‐β production, thereby inhibiting tumor progression by enhancing the recruitment of tumoricidal effector cells, especially NK cells.^72^ However, TAMs also enhance the production of eicosanoids through 15‐lipoxygenase‐2, which promotes the massive production of CCL2 and IL10, eventually leading to immune tolerance.^73^

We also systematically describe the regulation of TAM activity by the triglyceride hydrolysis pathway. Colorectal cancer‐associated TAMs have an increase of abhydrolase domain containing 5 (ABHD5), the cofactor of adipose triglyceride lipase, and a decrease of monoacylglycerol lipase (MGLL). ABHD5 in TAMs inhibited the accumulation of reactive oxygen species (ROS), which in turn reduced the production of C/EBPɛ‐dependent spermidine, and ultimately promoted the growth of colorectal cancer.^74^ MGLL deficiency in TAMs promotes M2 polarization through the CB2/TLR4 signal axis, inhibits the function of CD8^+^ T cells, and promotes the malignant progression of tumors.^75^ Actually, TAMs are also heterogeneous in lipid metabolism. We demonstrate that ABHD5 was heterogeneously expressed in TAMs. ABHD5‐deficient TAMs facilitate cancer metastasis by promoting the production of matrix metalloproteinases.^76^ In short, lipid metabolism of TAMs in the TME is changeable, which in most cases contributes to tumor progression.

### 3.4 Amino acid metabolism

The most studied amino acid metabolism of macrophages is arginine metabolism. In macrophages, L‐arginine has two main destinations.^77^ One is to generate NO through inducible NO synthase (iNOS). NO can suppress the key enzymes between the TCA and electron transport chain to inhibit oxidative phosphorylation, thus promoting glycolysis, which is the key feature of M1 macrophages. On the other hand, Arg‐1 converts L‐arginine into L‐ornithine. L‐Ornithine is further involved in the synthesis of proline and polyamine,^78^, ^79^ which is helpful for wound healing.^80^ This metabolic conversion is an important characteristic of M2 macrophages. In TAMs, decreased iNOS expression results in reduced production of NO,^81^ and increased expression of Arg1 may be related to hypoxia and high acidity of the TEM.^82^, ^83^ Although reduced NO leads to suppressed tumor cytotoxicity, Arg1‐primed TAMs promote M2 polarization and polyamine synthesis, thus enhancing tumor progression.^84^, ^85^ Furthermore, glutamine and tryptophan (Trp) also play important roles in TAMs. The expression of glutamine transporter and metabolic enzymes are significantly increased in TAM, which is beneficial for M2 polarization.^34^, ^86^ TAMs upregulate the indoleamine 2,3‐dioxygenase (IDO) and the rate limiting enzyme of Trp and inhibit the tumor immune response, thus promoting the malignant progression of tumors.^87^

### 3.5 T cells

After activation, T cells undergo huge genetic changes in a short period, forming different subpopulations to perform different functions. According to the secretion of cytokines and the expression of related proteins, CD4^+^ T cells can be divided into Th1, Th2, Th17, follicular helper T (Tfh), and Treg cells.^88^, ^89^ CD8^+^ T cells, once activated, are accompanied by a large amount of secretion of IFN‐γ and TNF‐a, and display strong cytotoxicity.^90^ However, persistent antigen exposure by tumors causes loss‐of‐function of T cells (eg, exhausted CD8^+^ T cells), resulting in tumor immune escape.^91^, ^92^, ^93^ The activation and function of T cells must be accompanied by metabolism changes.^94^ Here, we focus on studies that highlight links between metabolic and functional changes of T cells in the TME.

### 3.6 Glucose metabolism

Initially, T cells were thought to be prone to glycolysis after activation.^95^ For example, the differentiation of CD4^+^ T cells and CD8^+^ T cells is closely related to the activation of mTOR signaling, which is generally believed to promote glycolysis.^96^, ^97^ Glycolysis also promotes the secretion of IFN‐γ from T cells and enhances the function of CD8^+^ T cells.^98^ Recently, CD4^+^ cells and CD8^+^ T cells were found to have varying degrees of dependence on glycolysis and oxidative phosphorylation after activation.^98^ Whether pharmacological intervention of glycolysis or mitochondrial respiration inhibits the proliferation of CD4^+^ T cells and CD8^+^ T cells requires further studys.^99^, ^100^ The TME lacking glucose limits the aerobic glycolysis of tumor‐infiltrating T cells, thereby suppressing the tumoricidal effect.^101^ For example, low glucose reduces glycolytic flux by inhibiting Akt activity, thereby activating pro‐apoptotic B‐cell lymphoma‐2 (Bcl‐2) family members and inducing T cell apoptosis.^23^, ^102^ In ovarian cancer, cancer cells limit glucose metabolism by reducing the expression of T cell methyltransferase EZH2, thereby inhibiting T cell function and ultimately promoting tumor progression.^27^ In addition, tumor‐specific CD4^+^ and CD8^+^ T cells increase phosphoenolpyruvate (PEP) production by overexpressing phosphoenolpyruvate carboxykinase 1 (PCK1), thereby inhibiting sarco/ER Ca^2+^‐ATPase (SERCA) activity. PEP plays a new role in maintaining T cell receptor‐mediated Ca^2+^‐NFAT signaling and effector functions, and ultimately inhibits tumor growth.^24^ Moreover, acyl glycerol kinase (AGK) promotes glycolysis and antitumor activity of CD8^+^ T cells by inactivating PTEN and enhancing mTOR activity.^103^

### 3.7 Lipid metabolism

Naive T cells use oxidative phosphorylation to produce energy. Once T cells are activated to become effector cells, they start aerobic glycolysis to maintain their functions.^95^ When they become memory cells, they mainly restart to use oxidative phosphorylation, which requires fatty acid oxidation to produce more ATP for their own use.^104^ Memory T cells have a larger mitochondrial reserve, also called spare breathing capacity, than the naive T cells.^105^ Memory CD8^+^ T cells showed greater oxygen consumption and significantly enhanced fatty acid oxidation.^106^ When CD8^+^ T cells lack TNF receptor‐associated factor 6, a downstream signaling molecule of the TNF cytokine receptor, memory T cells cannot be formed.^107^ Moreover, TRAF6‐deficient T cells show defects in lipid oxidation, as expression of fatty acid metabolism genes is reduced in these cells.^107^ Besides, cholesterol metabolism is also highly associated with T cell activity. Yang et al claim that modulation of cholesterol metabolism by targeting the key cholesterol esterification enzyme ACAT1 can largely potentiate the antitumor response of CD8^+^ T cells.^108^

In the TME, increased Treg cells and exhausted CD8^+^ T cells cause the formation of an immunosuppressive microenvironment.^109^, ^110^ It has been found that Treg cells depend on lipid metabolism for their survival and function.^111^ It has also been reported that two different metabolites of LCA (3‐oxolca and isoalloLCA) regulate T cell function in mice, suggesting that bile acid metabolites directly regulate TH17 and Treg cell balance and host immunity.^112^ Recently, Field et al showed that fatty acid‐binding protein‐5 activates the IFN signal in Treg cells, thereby reducing the production of the regulatory factor IL‐10, which ultimately leads to the weakening of the immunosuppressive effect in TME.^111^

### 3.8 Amino acid metabolism

Amino acids play an important role in the maintenance of T cell phenotype and function. For example, IFNAR1, which is inherent in liver cells, inhibits the transcription of metabolic genes including Otc and Ass1, which in turn leads to a decrease in arginine concentration and an increase in ornithine concentration in the circulation, ultimately suppressing the activity of virus‐specific CD8^+^ T cell.^113^ In addition, a recent study found that amino acids enhance mTORC1 signal and Treg cells function through the small G proteins Rag and Rheb.^114^ In tumors, the availability of many amino acids is low in the TME, especially glutamine.^17^ Previous studies have found that ERK/MAPK‐coordinated regulation of glutamine uptake and metabolism is essential for T lymphocyte activation.^115^ Moreover, Trp is heavily utilized by tumor cells, thus resulting in the low concentration of Trp in the TME.^116^ However, activated T cell is extremely sensitive to the concentration of Trp in the peripheral environment, which triggers the effector T cell apoptosis.^117^ In addition, Kynurenic acid, a metabolite of Trp, acts as a ligand to activate arylhydrocarbon receptor and regulate CD8^+^ T cells, ultimately suppressing the antitumor immune response.^118^ The arginase in tumors and myeloid cells also causes extremely low arginine concentrations in the TME, inhibiting T cell activation and proliferation.^119^ For example, a latest study using proteomics, metabolomics, and other big data analysis shows that activated T cells consume large amounts of arginine and rapidly convert it into downstream products. L‐Arginine induces a metabolic shift from glycolysis to oxidative phosphorylation by transcription factors BAZ1B, PSIP1, and TSN, and promotes the survival and proliferation of memory T cells, thus enhancing the tumoricidal effect.^120^

### 3.9 NK cells

The NK cell is an important component of immune system. They are not only related to antitumor, antivirus infection, and immune regulation, but also in some cases participate in the occurrence of hypersensitivity and autoimmune diseases.^121^ In recent years, NK cells emerge as an important target for tumor immunotherapy because they kill tumor cells in different ways without the need for prior sensitization. However, hypoxia, high acidity, nutritional deficiencies, and immunosuppression of the TME change the balance between activation and inhibition of NK cells, ultimately limiting the function of NK cells.^122^ Moreover, the changes in glucose metabolism of NK cells play an important role in antitumor immunity.^123^, ^124^ Next, we discuss the metabolic effects of the TME on NK cells and their functional changes.

### 3.10 Glucose metabolism

The activation of NK cells depends on a wide range of signals through a series of receptors. Activated NK cells rapidly produce IFN‐γ to exert its effector functions, which depends on glycolysis and oxidative phosphorylation to supply energy.^125^, ^126^, ^127^ In addition, NK cells express three types of glucose transporters, GLUT1, GLUT3, and GLUT4, further illustrating the importance of glucose for NK cell activation.^128^, ^129^ Among these three glucose transporters, the increased expression of GLUT1 promotes glucose uptake and affects NK cell function.^130^ Moreover, sterol regulatory element binding proteins (SREBPs) regulate glycolysis and function of NK cells.^131^ Therefore, the lack of nutrition, especially glucose, in the TME affects the metabolism of NK cells and their tumoricidal effect. For example, in a mouse lung cancer model, the TME induces glucose metabolism disorder in NK cells, thereby leading to the loss of antitumor activity. In contrast, inhibition of FBP1, which is a key enzyme in the gluconeogenesis pathway and displays abnormally high expression in tumors, restores glycolysis and function of NK cells, ultimately inhibiting tumor progression.^28^ In addition, a large amount of TGF‐β in the TME inhibits mTOR, a key molecule that regulates cell metabolism and growth, and suppresses NK cell activity by affecting glucose metabolism.^132^, ^133^

### 3.11 Lipid metabolism

The changes in lipid metabolism are one of the most significant metabolic characters in both cancer cells and NK cells. SREBPs are a class of transcription factors that play central roles in lipid metabolism and control the expression of lipid synthesis‐associated genes.^134^ It has been found that the stimulation of cytokines (eg, IL‐2 and IL‐12) promoted the expression of SREBPs in NK cells, and might facilitate fatty acid and cholesterol synthesis through Fasn/Scd1 and Hmgcs1/Acat2, respectively.^131^ A recent study showed that high levels of cholesterol in the serum accelerate cholesterol accumulation in NK cells, resulting in the formation of lipid raft and the activation of immune signals. These changes ultimately enhance the cytotoxic activity of NK cells and inhibit the progression of liver cancer.^135^ Additionally, the metabolism of glycerol and phospholipids also play important roles in the activation of NK cells.^136^, ^137^ For example, diglyceride kinases (DGKs) control the level of DAG in cells through phosphorylating DAG into phosphatidic acid (PA). And DGKζ‐deficient NK cells release more IFN‐γ and enhance the tumor killing effect through ERK1 signaling.^137^ Adiponectin is a highly abundant hormone secreted by adipose tissue, which is involved in the metabolism of glucose and FA, and acts on various types of cells, including NK cells, which express high levels of AdipoRs, through adiponectin receptors 1 and 2 (AdipoR 1 and 2) and T‐cadherin.^138^, ^139^ Adiponectin might regulate the maturation and activation of NK cells.^139^ However, whether these effects are related to lipid metabolism and whether the TME can affect the activation of NK cells through this pathway require further investigation.

### 3.12 Amino acid metabolism

The utilization of amino acids in NK cells plays a key role in maintaining signaling pathways mediated by metabolic regulators (such as mTOR or cMyc).^140^, ^141^ For instance, arginine and glutamine affect mTOR signaling, and thus regulate the initial expression of cMyc. cMyc is a transcription factor, which is necessary in IL‐2/IL‐12‐induced metabolic and functional responses of NK cells in mice.^140^ In the TME, tumors and tumor‐related cells consume large amounts of amino acids such as arginine, Trp, and glutamine, leading to the accumulation of immunosuppressive metabolites.^142^ Among them, NO and L‐kynuric acid, respectively, inhibit the cytotoxic activity and proliferation of NK cells.^143^, ^144^ Therefore, in order to develop new treatment strategies, further study of the amino acids metabolism of NK cells in the TME and investigation of the correlation between metabolic changes and functions of NK cells are needed.

### 3.13 Dendritic cells

Dendritic cells (DCs), named for their nerve cell‐like dendritic morphologies,^145^ can be divided into bone‐marrow‐derived DCs (BMDCs) and plasmacytoid DCs (pDCs) according to their source, phenotype, and cytokine secretion.^146^ After dendritic cells sense pathogen‐associated molecular patterns or damage‐associated molecular patterns through pattern recognition receptors, they are activated from an immature tolerance status to mature immune stimulating phenotypes, and then use MHC Class I or II molecules to activate CD8^+^ or CD4^+^ T cells. This process is accompanied by changes in metabolic changes.^147^ It has been reported that immature resting BMDCs rely on fatty acid oxidation to perform oxidative phosphorylation in the mitochondrial electron transport chain to meet their energy requirements.^148^ Once activated, DCs increase the expression of a variety of molecules involved in antigen presentation, such as MHC molecules, cytokines, etc,^149^ which is accompanied by a sudden increase in glycolysis.^150^ Here, we discuss recent studies about how tumors manipulate DCs to interfere their homeostasis, and evade immune control by molecular pathways and metabolic changes.

### 3.14 Glucose metabolism

Activated DCs require high levels of glucose metabolism to meet their substances and energy requirements.^131^, ^151^ It has been reported that mouse BMDCs rapidly induce glycolysis through PI3k/AKT/mTOR/HIF‐1α signaling cascade after exposure to lipopolysaccharide, increasing the rate of glycolysis and lactic acid production.^152^, ^153^ Moreover, inhibition of BMDCs using glucose‐deficient media or glycolysis inhibitor, 2‐deoxyglucose, affects their activation and metastasis, including the expression of CD80, CD86, and CCR7, and the secretion of proinflammatory cytokines.^150^, ^151^ Importantly, the catabolism of prestored glycogen in mouse BMDCs is thought to be a key factor to drive TLR‐activated glycolysis.^154^ Thus, metabolic competition and glucose restriction in the TME affect the metabolism and function of DCs.^149^ For example, the low availability of glucose may interfere with glucose uptake or metabolism, thereby hindering protein glycosylation in the endoplasmic reticulum of tumor‐associated DCs (TADCs) and triggering an immunosuppressive endoplasmic reticulum stress response.^155^ Moreover, rapamycin‐mediated inhibition of mTOR can prolong the lifespan and mitochondrial activity of BMDCs stimulated by lipopolysaccharide, which may be involved in glucose metabolism.^156^

### 3.15 Lipid metabolism

DC maturation mediated by TLR signal requires activation of downstream signal transduction and metabolic changes. De novo lipid biosynthesis is an important metabolic process after BMDCs activation.^157^ Early glycolytic bursts after TLR sensing in BMDCs promote the production of citrate and acetyl CoA through TCA, which is a substrate for lipid synthesis.^150^ Recent studies have also shown that p32 positively regulates the synthesis of citrate and lipid, ultimately facilitating the maturation and activation of DCs.^158^ In contrast, the DCs from solid tumors contain lots of cellular lipid droplets, with a defect in antigen presentation and subsequent T cell activation.^159^ A later study have shown that lipid bodies containing oxidatively truncated lipids, but not the lipid bodies in normal cells, block antigen cross‐presentation by DCs in cancer.^160^ Inhibition of fatty acid synthesis restore the function of DCs and T cells, thereby inhibiting tumor progression.^160^ Thus the regulation of DC activation is a complicated process, involving reprogramming of signal transduction and lipid metabolism. For example, the Wnt signaling can integrate PPARγ‐regulated fatty acid oxidation, driving DC tolerization, Treg recruitment, and immune evasion.^161^ Moreover, the retinoic acid derived from vitamin A metabolism also promoted Treg activation and tumor progression.^162^

### 3.16 Amino acid metabolism

Amino acid metabolism is suggested as an important node of immune regulation.^163^, ^164^ For example, IDO‐1, the rate‐limiting enzyme of kynurenine pathway, catalyzes an essential amino acid L‐tryptophan, leading to Trp depletion and the production of a series of immunoregulatory molecules collectively known as kynurenines. IFN‐γ‐stimulated DCs have an increase of IDO‐1 expression and activity.^165^ Thus the effector T cells activated by DCs might suppress DCs’ function as a negative feedback. Actually, IDO‐1 in DCs can be induced by multiple factors, such as TGF‐β, IL‐32, and other cytokines derived from tumor cells, other immune cells, or even the DCs themselves in the TME.^166^, ^167^, ^168^ Those findings integrate the cytokine signals with the Trp metabolism and immune suppression in the TME. Similarly, Arg‐1 is another immunoregulatory enzyme catalyzing the degradation of L‐arginine. A series of Th2 cytokines such as IL‐4, IL‐13, and TGF‐β can induce Arg‐1 expression in myeloid cells, including macrophages and DCs.^169^, ^170^ Notably, TGF‐β can stimulate IDO‐1 and Arg‐1 expression simultaneously in DCs, indicative of an intensive immune suppression. Moreover, Arg1 activity is absolutely required for IDO1‐dependent signaling events as initiated by TGF‐β. DCs can be conditioned by Arg1^+^ MDSCs to express an IDO1‐dependent immunosuppressive phenotype.^171^ Consistently, arginine‐depleted TADCs inhibit CD8^+^ T cell proliferation and IFN‐γ secretion.^172^ Further investigation of amino acid metabolism in TADCs is extremely important for understanding TADCs activation and developing potential therapeutic strategies.

### 3.17 Cancer immunotherapy

Cancer immunotherapy is a type of tumor treatment that reactivates the body's antitumor immunity by regulating the immune system. It includes immune checkpoint inhibitors (ICIs), T‐cell transfer therapy, monoclonal antibodies, cancer vaccines, and immune system modulators.^173^, ^174^, ^175^ Immunotherapy appears better than conventional chemotherapy at treating some forms of cancer in patients, especially advanced patients, so it has attracted intensive attentions from researchers in recent years. The positive response of immunotherapy usually relies on the interaction of tumor cells with immunomodulation in the TME. Therefore, the TME plays an important role in suppressing or enhancing the immune response. Understanding the interaction between immunotherapy and the TME is not only the key for analyzing the mechanisms of tumor progression, but it is also of great significance to provide new methods for improving the efficacy of current immunotherapy. Next, we focus on the current types of immunotherapy and their latest developments.

### 3.18 Immune checkpoint inhibitors

ICIs are monoclonal antibodies that bind to immune checkpoints to stop tumors from inhibiting T cells, including anticytotoxic T lymphocyte antigen‐4 (CTLA‐4), antiprogramed cell death protein 1 (PD‐1), and anti‐PDL‐1.^176^ Under normal circumstances, CTLA‐4 and PD‐1 signals are strictly regulated to allow self‐tolerance; however, tumor cells can use these pathways to evade the immune response and establish a microenvironment conducive to tumor growth.^177^, ^178^ ICIs can reactivate the immune system and prevent tumor immune escape.^179^ Currently, a variety of checkpoint inhibitors have been used in a variety of clinical oncology, and achieved good results, especially in melanoma and other malignant tumors.^180^, ^181^, ^182^, ^183^ However, a large proportion of patients are either insensitive to ICIs or are burdened by adverse side effects, including dermatologic toxicity, gastrointestinal toxicity (diarrhea or colitis, hepatitis), endocrinopathies (thyroid toxicity, thyroid toxicity), pneumonitis, and rare immune‐related adverse events, during treatments^184^, ^185^ (Table 1).

**TABLE 1:** PD‐1 and PD‐L1 blocking agents in tumor therapy

Tumor type	Target—antibody	Response rate
Melanoma	PD‐1—Nivolumab	CR 8.9%, PR 41%^257^
		CR 4%, PR 30%^258^
		CR 3.3%, PR 28.3%^259^
	PD‐1—Pembrolizumab	CR 5‐6%, PR 27‐29%^260^
		CR 2‐3%, PR 19‐23%^261^
Hodgkin's lymphoma	PD‐1—Nivolumab	CR 14%, PR 55%^262^
	PD‐1—Pembrolizumab	CR 22%, PR 47%^263^
NSCLC	PD‐1—Nivolumab	CR 0.7%, PR 19.3%^264^
		CR 1.4%, PR 17.8%^265^
	PD‐1—Pembrolizumab	ORR 18‐19% (PD‐L1 > 1%), 29‐30% (PD‐L1 > 50%)^266^
		Hazard ratio 0.53 versus chemotherapy alone^267^
		CR 4%, PR 41%^268^
	PD‐L1—Atezolizumab	OS 12.6 m versus 9.7 m in chemotherapy arm^269^
	PD‐L1—Durvalumab	Hazard ratio 0.52 versus placebo^270^
MSI‐H and dMMR CRC	PD‐1—Nivolumab	CR 2.7%, PR 30%^271^
Gastric cancer	PD‐1—Pembrolizumab	ORR 13.3% in PD‐L1 positive^272^
	PD‐1—Nivolumab	ORR 18.7%, DCR 31.2%^273^
Advanced Endometrial Cancer	PD‐1—Pembrolizumab + Lenvatinib	ORR 63.6%^274^
HNSCC	PD‐1—Nivolumab	OS 7.5 versus 5.1 m for investigator's choice^275^
	PD‐1—Pembrolizumab	CR 5%, PR 11%^276^
Urothelial carcinoma	PD‐1—Nivolumab	CR 2.6%, PR 17%^277^
	PD‐1—Pembrolizumab	ORR 21%^278^
		ORR 28.6%^279^
	PD‐L1—Atezolizumab	CR 6.7%, PR 16.8%^280^
		CR 5.5%, PR 9.4%^281^
	PD‐L1—Durvalumab	CR 2.7%, PR 14.3%^282^
	PD‐L1—Avelumab	CR 5.6%, PR 10.6% (at 6 months follow‐up)^283^
Merkel cell carcinoma	PD‐L1—Avelumab	CR 11.4%, PR 21.6%^284^
Gastroesophageal Cancer.	PD‐1—Nivolumab + 5‐Fluorouracil	DCR 73.3%, OS 13.3 m^285^
MSI‐H and dMMR solid tumors	PD‐1—Pembrolizumab	ORR 39.6%^286^
HCC	PD‐1—Nivolumab	ORR 14.3%^287^
nccRCC	PD‐1—Nivolumab	ORR 18.6%, DCR 53.4%^288^

Abbreviations: CR, complete response; CRC, colorectal cancer; DCR: disease control rate; dMMR, mismatch repair deficient; HCC, hepatocellular carcinoma; HNSCC, head and neck squamous cell carcinoma; MSI‐H, microsatellite instability high; nccRCC, nonclear cell renal cell carcinoma; NSCLC, nonsmall cell lung cancer; ORR, overall response rate; OS, overall survival; PR, partial response.

In order to increase the responsive rate of ICIs, some combination therapies have been developed. Among them, combination of CTLA‐4 and PD‐1 blockers has already proved to be highly effective in clinical trials.^186^ In addition, many factors have been identified to cause the insensitivity to ICIs treatment. For example, macrophages are also important in targeting the PD‐1/PD‐L1 axis. Macrophages remove anti‐PD1 antibodies from T cells, attenuating the response of T cells,^187^ and meanwhile, express PD1 on their surface, thereby weakening their phagocytic activity.^188^ In response to the toxic side effects of ICIs, steroids and immune‐modulating therapy have been reported to have good effects.^189^ Tokunaga et al showed that early administration of corticosteroid, rather than late administration of corticosteroid, led to tumor regeneration, suggesting that early administration of corticosteroid inhibited memory CD8^+^ T cells, which is associated with persistent antitumor responses.^190^

### 3.19 T cell transfer therapy

T cell transfer therapy is an immunotherapy that uses a patient's own immune cells to attack cancer cells.^191^ There are two main types: tumor infiltrating lymphocyte (TIL) therapy and CAR‐T cells therapy.^192^, ^193^ Both methods require collecting immune cells from cancer patients, culturing them in vitro, and then injecting them back into patients via intravenous injection.^194^ T cell transfer therapy is also called adoptive cell therapy, adoptive immunotherapy, and immune cell therapy.^195^

In TIL therapy, researchers isolated special lymphocytes that can recognize tumor cells, and reinfused to patients to treat tumors after rapid and massive expansion of these cells.^196^ Although TIL therapy is effective in some patients with melanoma and has achieved good results in other cancers such as cervical squamous cell carcinoma and bile duct cancer, this treatment is still in the experimental stage.^197^, ^198^ CAR‐T cell therapy is designed in vitro to make the obtained T cells produce a protein called CAR, also known as a chimeric antigen receptor.^194^ CAR can promote T cells to attach to specific proteins on the surface of cancer cells, increasing their ability to attack cancer cells.^194^, ^199^ CAR‐T is currently used to treat hematological malignancies.^200^, ^201^

However, CAR‐T cells can induce a large number of adverse side effects.^202^ For example, the most common toxic reactions in hematological malignancies are cytokine release syndrome (CRS) and ICANS.^202^ Recently, it has been reported that NK cells modified to express anti‐CD19 CAR not only overcome the toxic effect of anti‐CD19 CAR‐T cells, but also respond well to treatment.^203^ In addition, Correia et al designed a chemically destroyable heterodimer (CDH), which can be inactivated by small molecule compounds, based on the binding of two human proteins on CAR‐T cells.^204^

### 3.20 Monoclonal antibodies

Monoclonal antibodies are antibodies that bind to specific antigens on cancer cells so that they can be better detected and destroyed by the immune system.^205^ Some monoclonal antibodies also help the immune system to fight against cancer cells.^206^, ^207^ Recently, Yang et al designed tri‐specific antibodies against CD38, CD28, and CD3, which could significantly enhance the activation of T cells and the recognition and killing of tumor cells, and have achieved good results in animal models.^208^ In addition to targeting T cells, macrophages are also good effector cells. CD24 has recently been identified to regulate macrophage phagocytic function through siglecl‐10 signaling, affecting tumor progression.^209^ CD47 also plays a role in regulating macrophage phagocytosis.^210^ Several monoclonal antibodies against CD47, including Hu5F9‐G4 and ALX148, have been shown encouraging data in preclinical trials.^211^, ^212^

### 3.21 Others

Cancer vaccines are currently mainly divided into preventive vaccines and therapeutic vaccines.^213^ The former ones are used to prevent cancer by preventing and killing certain viral or bacterial infections. For example, cervical cancer vaccine can prevent HPV infection, thus preventing cervical cancer.^214^ The latter ones are used to control or kill tumor cells by activating body‐specific immune functions through cancer cell antigens.^213^ The first FDA‐approved cancer treatment vaccine is talimogene laherparepvec (T‐VEC), also known as oncolytic virus treatment.^215^ The virus can infect cancer cells and normal cells. Normal cells can kill the virus, whereas cancer cells cannot.^216^ Recently, Keskin et al have demonstrated that a multi‐epitope, personalized neoantigen vaccination strategy is feasible in the treatment of glioblastoma patients. This strategy has been tested in high‐risk melanoma patients.^217^

Immunomodulator is an immunotherapy that enhances the body's immune response to cancer, including cytokines, Bacillus Calmette‐Guerin (BCG), immunomodulatory drugs, and so forth.^218^, ^219^, ^220^ It is generally used to assist other tumor treatments. For example, IL‐2, also known as T‐cell growth factor, increases the number of white blood cells such as NK and T cells in the body, and then causes an immune response to cancers.^221^ In a multicenter, randomized phase 3 clinical trial, dinutuximab beta combined with IL‐2 was found to be more effective in treating neuroblastoma than dinutuximab beta alone.^222^

## 4 METABOLISM AND CANCER IMMUNOTHERAPY

**FIGURE 3:**
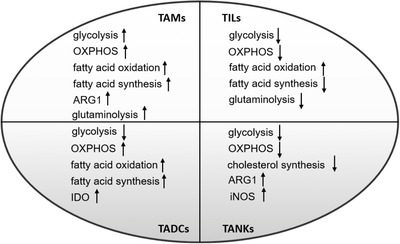
Metabolic changes of tumor‐associated immune cells *Note*. “↑” indicates “increase,” and “↓” indicates “decrease.” Abbreviations: OXPHOS, oxidative phosphorylation; TADCs, tumor‐associated dendritic cells; TAMs, tumor‐associated macrophage; TANKs, tumor‐associated natural killer cells; TIL, tumor‐infiltrated lymphocytes.

Although a large number of clinical trials of tumor immunotherapy have achieved great success by enhancing host immunity, this is limited to a small number of patients.^184^ Increasing evidence indicates that tumor and its interstitial cell metabolic reprogramming plays an important role in tumor immunosuppressive response and resistance to immunotherapy.^142^ In the TME, the metabolism of immune interstitial cells is at a disadvantage because tumor cells consume a large amount of nutrients and increase inhibitory signals.^142^ At the same time, nutritional deficiencies and metabolic wastes accumulate in TME, leading to metabolic conversion of immune interstitial cells, which impairs their proliferation and function.^223^ Therefore, an in‐depth understanding of the metabolic changes in TME and their impact on immune cell metabolism may help to find new and promising ways to rebuild the metabolism of immune cells and thus promote existing immunotherapy. Based on aforementioned introductions, we summarized the main metabolic changes of tumor‐associated macrophages, T cells, dendritic cells, and NK cells (Figure 3). Next, we would conclude the effects of metabolic intervention on tumor cells and immune cells, especially on T cell‐based immunotherapy.

### 4.1 Tumor metabolism and cancer immunotherapy

As we mentioned earlier, in tumors, despite the presence of oxygen, cancer cells still show more glycolysis, because glycolysis is much faster than OXPHOS, which provides cancer cells with a competitive advantage and makes them consume more glucose than surrounding slowly dividing cells and grows under conditions of hypoxia and nutritional deficiencies.^224^ Therefore, there have been several drugs targeted at tumor aerobic glycolysis for cancer treatment, including 2‐Deoxyglucose^225^ (the inhibitor of GLUT1 and HK), Dichloroacetate^226^ (inducing a shift from glycolysis to OXPHOS), and the inhibitor of phosphofructokinase 1.^227^ However, these drugs also significantly inhibit T cell function and promote immunosuppression.^228^ Therefore, targeting tumors is important for the application of glycolysis inhibitors in tumor therapy. In addition, because a large amount of lactic acid secreted by aerobic glycolysis of cancer cells leads to the formation of acidic immunosuppressive TME,^31^ some drugs targeting lactic acid to treat tumors are also being developed, including targeting lactate dehydrogenase (LDH), monocarboxylate transporter (MCT) inhibitors, and oral bicarbonate supplementation.^229^ Lenalidomide, a new MCT disruptor, has been found to enhance T cell IL‐2 and IFN‐γ secretion while inhibiting tumor cell proliferation.^230^ Diclofenac, a nonsteroidal anti‐inflammatory drug, can inhibit glioma growth, Treg infiltration, and lactic acid secretion.^231^ Oral bicarbonate neutralizing lactic acid combined with anti‐PD‐1 immunotherapy can inhibit tumor growth in melanoma model, and combined with adoptive T cell transfer can prolong mouse survival.^232^ Recently, Professor Liu and his colleagues proposed a novel intracellular/extracellular lactic acid depletion strategy that can be used in conjunction with immunotherapy to combat tumors.^35^ Therefore, the application of targeting lactate should be feasible to improve the effect of immunotherapies.

In addition to glucose metabolism, tumor cells also consume large amounts of amino acids for their own advantage. Therefore, targeting these amino acids (such as L‐arginine, Trp, and glutamine) has broad application prospects in tumor treatment. It has been reported that some malignant tumors lack the arginine succinate synthase, a key enzyme of the urea cycle, causing them to use in vitro amino acids to meet their own growth needs.^233^ Therefore, arginine deprivation therapy has been invented and has been used in a variety of tumor treatments, including advanced melanoma, pancreatic tumor, prostate cancer, stromal tumor, hepatocellular carcinoma, and lymphoma.^234^ Notably, CB‐1158, an ARG inhibitor, in combination with immunotherapy has achieved good results in clinical trials and can block myeloid‐mediated immunosuppression in the tumor microenvironment.^235^ In addition, eliminating IDO to eliminate the role of Trp in tumor promotion and immunosuppression is also a key target for tumor immunotherapy, such as epacadostat^236^ and indoximod.^237^ The inhibitors of IDO, which can directly inhibit IDO activity to inhibit Trp degradation, enhance cytotoxic T cell function and reduce the number of Tregs, or interfere with Trp degradation signals, and avoiding T cells immunosuppression.^238^ Therefore, IDO may also be one of the key targets for immunotherapy. Glutamine is one of the main sources of energy for tumor cells.^17^ Glutamine analogs^239^ (6‐diazo‐5‐oxo‐L‐norleucine, azaserine, and acivicin) and glutamine transporter inhibitors^240^ (gamma‐lglutamyl‐p‐nitroanilide and benzylserine [H‐Ser (Bzl) ‐OH]) have been found to suppress tumor progression in pre‐ and clinical trials. Interestingly, Leone et al found that JHU083, a precursor of DON (the inhibitor of glutaminase), can affect cancer cells without affecting normal cells, making it unable to use glutamine and inhibit tumor progression.^241^ At the same time, JHU083 can also enhance antitumor immunity by infiltrating CD8^+^ T cells, and combined with anti‐PD‐1 treatment showed more significant antitumor effects.^241^ In addition to the above mentioned, there are several metabolic interventions in combination with immunotherapy to treat tumors (Table 2)

**TABLE 2:** Ongoing trials of metabolic interventions combined with immune‐checkpoint inhibitors

Pathways	Metabolic agent	Immunotherapy	Cancer types	Study phase	ClinicalTrials.gov references
Inhibitors of glucose metabolism	Metformin (various effects on glucose levels and metabolism)	Pembrolizumab	Advanced‐stage melanoma	I	NCT03311308
		Nivolumab	Unresectable or metastatic NSCLC	II	NCT03048500
Glutamine and glutamate pathway inhibitors	CB‐839	Nivolumab	Advanced‐stage clear cell RCC, melanoma, or NSCLC	I/II	NCT02771626
	Trigriluzole	Nivolumab or pembrolizumab (anti‐PD‐1 antibodies)	Metastatic or unresectable solid tumors or lymphom	II	NCT03229278
Arginine pathway inhibitors	INCB001158 (arginase inhibitor)	Pembrolizumab	Advanced‐stage solid tumors	I/II	NCT02903914
	ADI‐PEG 20 (PEGylated arginine deiminase)	Pembrolizumab	Advanced‐stage solid tumors	I	NCT03254732
		Atezolizumab (anti‐PD‐L1 antibody) + pemetrexed and carboplatin	Advanced‐stage NSCLC	I	NCT03498222
IDO inhibitors	Epacadostat (INCB024360; IDO1 inhibitor)	Pembrolizumab	Ovarian clear cell carcinoma	II	NCT03602586
			Small‐cell lung carcinoma	II	NCT03402880
			Endometrial carcinoma	II	NCT03310567
			Gastrointestinal stromal tumors	II	NCT03291054
			Urothelial cancer	III	NCT03361865
			HNSCC	II	NCT03325465
		INCAGN01876 (agonistic anti‐GITR antibody) + pembrolizumab	Advanced‐stage cancers	I/II	NCT03277352
		Nivolumab	Glioblastoma	I	NCT03707457
	Linrodostat (BMS‐986205; IDO1 inhibitor)	Relatlimab (anti‐LAG3 antibody) and nivolumab	Advanced‐stage cancers	I/II	NCT03459222
		Nivolumab	Advanced‐stage cancers	I	NCT03335540
	Indoximod (IDO1 and IDO2 inhibitor)	Pembrolizumab or nivolumab	Advanced‐stage melanoma	II/III	NCT03301636
		Ipilimumab (anti‐CTLA‐4 antibody), nivolumab, or pembrolizumab	Metastatic melanoma	I/II	NCT02073123
	Navoximod (GDC‐0919 or NLG919; IDO1 inhibitor)	Atezolizumab	Advanced or metastatic solid tumors	I	NCT02471846
	HTI‐1090 (SHR9146; dual IDO1‐TDO inhibitor)	Camrelizumab (SHR‐1210; anti‐PD‐1 antibody) ± apatinib (VEGFR TKI)	Advanced‐stage solid tumors	I	NCT0349163
	LY3381916 (IDO1 inhibitor)	LY3300054 (anti‐PD‐L1 antibody)	Advanced‐stage solid tumors	I	NCT03343613
Inhibitors of COX enzymes and/or PGE2 signaling	Aspirin (COX1 and/or COX2 inhibitor) or celecoxib (COX2 inhibitor)	BAT1306 (anti‐PD‐1 antibody)	Advanced‐stage MSI‐H/dMMR cancers	II	NCT03638297
	Aspirin	Pembrolizumab + clopidogrel (P2Y12 inhibitor)	Recurrent or metastatic HNSCC	I	NCT03245489
	Grapiprant (EP4 antagonist)	Pembrolizumab	NSCLC	I/II	NCT03696212
		Pembrolizumab	Advanced‐stage or progressive microsatellite‐stable CRC	I	NCT03658772

Abbreviations: COX, cyclooxygenase; CRC, colorectal cancer; EP4, prostaglandin E2 receptor 4; GITR, glucocorticoid‐induced TNFR‐related protein; HNSCC, head and neck squamous cell carcinoma; MSI‐H/dMMR, microsatellite instability‐high and/or mismatch repair‐deficient; LAG3, lymphocyte activation gene 3 protein; NSCLC, nonsmall‐cell lung carcinoma; P2Y12, P2Y purinoceptor 12.

### 4.2 T cell metabolism and cancer immunotherapy

Immunotherapy‐mediated enhancement of tumor‐specific T effector cells mostly has transient antitumor effects. Because metabolism plays an important role in homeostasis and adaptation under intracellular and extracellular stimulation, the combination of metabolism‐targeting drugs with immunotherapy may form a more promising treatment. This may promote the production of T memory cells with enhanced activity and plasticity in order to differentiate the effector cells when re‐exposed to cancer antigen.

Recent evidence suggests that both checkpoint ligation and inhibition may directly alter the metabolism and characteristics of T cells and cancer cells. For example, the binding of PD‐1 to its ligand can affect TIL metabolism by inhibiting glycolysis and upregulating FAO.^242^ Similarly, the signals received by CTLA‐4 and B7 can inhibit glycolysis.^242^ In addition, purely targeted metabolism may affect multiple immune cell populations and may have unpredictable results on systemic antitumor effects. For example, effector T cell proliferation and differentiation depend on FAS, and FAO is essential for the development of CD8^+^ T cell memory cells and the differentiation of CD4^+^ Treg cells.^243^, ^244^ Therefore, it is extremely important to explore more reasonable therapeutic methods of targeted metabolism combined with immunotherapy. It has been reported that targeting AGK in CD8^+^ T cells can enhance glycolytic metabolism levels to promote antitumor activity of CD8^+^ T cells.^103^ Similarly, Zhang et al also found that in the face of TME with low oxygen and low glucose, T cells lack glucose supply and mainly carry out fatty acid metabolism capacity. Moreover, the use of PPARα agonists not only enhances the fatty acid metabolism and anti‐tumor function of T cells, but also enhances the therapeutic effect in combination with PD‐1 inhibitors.^245^ These all provide new ideas for how to target T cell metabolism and regulate its antitumor function.

In addition to the abovementioned, adenosine 5′‐monophosphate‐activated protein kinase (AMPK), as a key molecule in the regulation of biological energy metabolism, is also a key target molecule in the regulation of tumor immune metabolism.^246^ Metformin, an AMPK activator, can promote the differentiation of CD8^+^ memory T cells and may protect cell apoptosis and enhance antitumor effects.^247^ At the same time, metformin can also enhance the antitumor effect of PD1/CTLA‐4 blockade by reducing tumor hypoxia.^248^, ^249^ However, similar studies have found that metformin can promote the formation of Treg cells and inhibit Th1 and Th17, reducing the effect of tumor treatment.^244^ Therefore, because of the characteristics of cancer and the type of immune cells that dominate TME in each cancer type, the therapeutic effects obtained by targeting AMPK are also different. Solving this problem may also be a good immune metabolic target for tumor treatment point.

Similarly, improving metabolic impairment caused by immunotherapy through metabolic regulation is also a method. PD1/PDL‐1 can inhibit mitochondrial function of T cells, and then affect its activity.^250^ Moreover, mitochondrial metabolites ROS have also been shown to activate CD4^+^ and CD8^+^ T cells.^251^ And it has been found that mitochondrial activating chemicals (ROS precursors or mitochondrial uncouplers) and PD‐1 block synergistically enhance T cell‐dependent antitumor activity.^252^ However, because targeting mitochondria often leads to strong toxic and side effects, this requires further investigation.

### 4.3 Others

We mentioned above that not only exhausted CD8^+^ T cells and Treg cells, but M2 TAM, TADC, and NK cells also participate in the formation of immunosuppressed TME. Therefore, it is important to explore these metabolic changes in immune cells and their impact on immunotherapy. TAMs account for the largest proportion of tumor interstitial immune cells and must undergo certain metabolic reprogramming to survive in the tumor microenvironment. At present, metabolic reprograming of TAMs to promote their transition to M1 type has become an important antitumor strategy for targeting macrophages.^50^ Our previous research has found that the key molecules that regulate the fatty acid metabolism of macrophages can promote their M1 polarization, maintain the activity of CD8 + T cells, and then inhibit tumor progression.^74^, ^75^ The enzyme PI3Kγ, important for targeted phospholipid metabolism, can promote macrophage reprogramming and enhance T cell response, which can be used as a single drug or combined with T cell checkpoint blockade to inhibit tumor progression.^253^, ^254^ In DCs, the combination of FAO inhibition and anti‐PD‐1 blockade indicates that host survival has been significantly improved, driven by enhanced antitumor immunity.^161^ In addition, aspirin, a nonsteroidal anti‐inflammatory drug that blocks the COX‐1/2 pathway, and celecoxib, a COX‐2 inhibitor, can limit the production of PGE2 and interfere with lipid metabolism, and combine anti‐PD‐1 therapy can further enhance the antitumor effect.^255^ NK cells regulate T cell activity through the PD1/PDL1 axis, and NK cells in TME also undergo large tumor‐promoting metabolic changes, so targeting NK cell metabolism may also provide new ideas for tumor immunotherapy.^256^

## 5 CONCLUSION

This review introduces the characteristics of TME, the metabolic characteristics of immune cells, and the progress of tumor immunotherapy. Facing the dilemma of tumor treatment, immunotherapy gives new hope to tumor patients. However, current immunotherapies, such as CAR‐T, are only effective in a small number of patients with solid tumors. This phenomenon suggests that comprehensive treatment may be the way to deal with tumors. A large number of previous studies have confirmed that the TME plays a key role in the tumor progression and treatment response. Metabolic reprograming is a main feature of tumor microenvironment. From the perspective of metabolic intervention, improving the immune status of tumor microenvironment is expected to provide promising strategies for enhancing the therapeutic effect of tumors. However, up to now, the understanding of tumor microenvironment is still in a relatively preliminary stage. Future studies need to focus on the original driving forces for the formation of TME, variation of TME between different tumor types, ontogeny of the immune cells in TME, the metabolism and immune heterogeneity of tumor‐related immune cells, the crosstalk between immune cells and tumor cells, and the effects of metabolic reprogramming on T cell therapies.

Of course, some problems need to be solved quickly. First of all, exploring the overlapping mechanisms of primary and secondary immune escape may be in dire need. As mentioned above, although some tumor patients respond well to tumor immunotherapy, a large number of patients are not sensitive to treatment. Importantly, some patients will eventually see their tumors return even if they respond to treatment. The former is called primary immune escape and the latter is called secondary immune escape. The two immune escape mechanisms overlap and immune metabolic changes are likely to be involved. Exploring this overlapping mechanism is crucial to rapidly improve the effect of cancer immunotherapy and improve the patients’ quality of life, which may be one of the important directions of tumor immunotherapy in the future. Moreover, targeting the role of immunometabolism in therapy resistance is also an urgent and interesting direction. Many studies have found that drug resistance induced by traditional tumor therapy is closely related to changes in tumor immunometabolism, and reprogramming tumor immunometabolism is crucial to restore the therapeutic effect. Clarifying these questions will help us to identify key pathways and targets that will ultimately serve the cancer therapy in the clinic.

## CONFLICT OF INTEREST

The authors declare no conflict of interest.
